# Antiseptic Dressings

**Published:** 1889-08-10

**Authors:** 


### New Preparations.

### ANTISEPTIC DRESSINGS.

It is almost enough to make a physician turn surgeon to see the marked and wonderful progress that pure surgery has made during the last quarter of a century. Sir Joseph Lister, intense, resolute, conscientious, courageous, scientific, has lived to initiate and to see triumphant as complete a revolution in the surgical art as was the change from a Stuart dynasty to constitutionalism under William of Orange. The Macaulay of the surgical art has yet to arise; but when he does appear he will tell the world a story of triumphs which will compare not unfavourably with the onward march of political freedom, or the progress of general science in these last decades.

A red box with a glass lid, twelve inches long by six wide and three deep, filled with various lints, catgut, bandages, and tows, is what seems to remain of Listerism after thirty years' hard study and stiff fighting against prejudice, selfishness, and stupidity. This is all that many men, even surgeons and hospital surgeons, see in the work to which Lister has devoted his life. It does not seem very wonderful. Nor is it wonderful to a person who is more astonished by a display of fireworks than by the germination of an acorn and the growth of an oak. But to any one who understands the germ theory, now demonstrated to be the science of germ life and activity, the little box represents the greatest advance in scientific and practical medicine and surgery which has been made since the birth of the medical art. It stands for the whole germ and bacillary pathology, and for the whole of the antiseptic practice of the medical and surgical arts. So wide-reaching and potential for change and progress is a sound principle in the hands of an enthusiastic and far-seeing disciple and teacher of truth.

This box of antiseptic dressings has been submitted to us, and our opinion thereupon is solicited. The present writer is an old student of Sir Joseph Lister's, and well remembers both his clinical lectures, his surgical practice, and the complicated dressings he used to employ. He remembers also the pride with which a dressing that had been applied to a wound for days without removal, and which under a former system would have been a plaster of dangerous and offensive poison, was removed by the dresser at Mr. Lister's bidding, and both the wound and the dressing shown to be as pure and sweet as on the first day of the operation. These old and complicated dressings were identical in principle with the simple and convenient applications of to-day. At Milne's antiseptic factory at Ladywell—where the new dressings are prepared according to Sir Joseph Lister's own special instructions, *e.g.*, the carbolised catgut, the prepared tow, the gauze, also carbolised, the carbolic bandages, to which have been more recently added the boracic lint, the iodo-form gauze, and other specialties—there may be seen and procured all the fully developed products of which the rougher dressings of twenty years ago were the rude and embryonic beginnings. Their surgical value it would be superfluous to dilate upon. That is admitted on all hands, and is beyond question or doubt. What we may call special attention to is their neatness, convenience, and general excellence from the standpoint of the clinical clerk and dresser. Who remembers "the long, felted, unmanageable carbolised tow" of former days? Let him compare it with the neatly cut and "ready-for-use" article of the present time. That will give him at once the contrast between the whole series of dressings of the olden times and those of to-day.

Practically, everything that the surgeon or dresser requires is prepared antiseptically by Mr. Milne at his Ladywell factory. Not only so, but Surgeon-Major Bourke's first field dressing and various antiseptic specialties used by the physician and the accoucher are also there manufactured. It is undoubted that they are all of the best. Sir Joseph Lister's connection with their preparation is abundant guarantee for that. We do not hesitate to say that such antiseptic preparations and dressings as those prepared at Ladywell should be in every hospital in the world, whether large or small, civil or military, general or special. And if the ordinary practitioner, in however remote a country town he may be located, really desires to do his duty to his patients, he, too, will keep always on hand a selected stock of these indispensable requisites. Nowhere can they be obtained better or cheaper than at Mr. Milne's Ladywell factory.

